# An unusual case of neuroblastoma presenting with prolonged watery diarrhea in a pediatric patient

**DOI:** 10.11613/BM.2025.020901

**Published:** 2025-06-15

**Authors:** Claire Claeyssens, Peter Witters, Heidi Segers, Jan De Koster, Elena Levtchenko, Pieter Vermeersch

**Affiliations:** 1 Clinical Department of Laboratory Medicine, University Hospitals Leuven, Leuven, Belgium; 2 Clinical Department of Pediatrics, University Hospitals Leuven, Leuven, Belgium; 3 Department of Development and Regeneration, KU Leuven, Leuven, Belgium; 4 Department of Oncology, KU Leuven, Leuven, Belgium; 5 Clinical Department of Pediatrics, Ziekenhuis Oost-Limburg, Genk, Belgium; 6 Department of Development & Regeneration, KU Leuven, Belgium; 7 Department of Cardiovascular Sciences, KU Leuven, Belgium

**Keywords:** neuroblastoma, acidosis, hyperaldosteronism, hypokalemia, catecholamines

## Abstract

Neuroblastomas represent a diverse group of neuroblastic tumors characterized by variability in their clinical progression and degree of differentiation. In rare cases, patients with neuroblastoma may present with paraneoplastic syndromes, such as watery diarrhea, hypokalemia, and achlorhydria (WDHA syndrome), linked to the secretion of vasoactive intestinal peptide (VIP). We report a case of a 14-month-old girl presented with a three-week history of watery diarrhea and signs of dehydration with no other symptoms. The patient’s medical history was unremarkable, and no medication use was reported. Venous blood gas analysis revealed a normal anion gap metabolic acidosis with severe hypokalemia. The patient was referred to our hospital 48 hours post-admission due to persistent hypokalemic metabolic acidosis, unresponsive to intravenous fluid therapy. The primary causes of normal anion gap metabolic acidosis in young children are gastrointestinal bicarbonate loss due to diarrhea and renal bicarbonate loss. Semi-quantitative urine organic acid analysis, reported 48 hours after admission, revealed increased vanillylmandelic acid (VMA) (89 mmol/mol creatinine) and homovanillic acid (HVA) (21 mmol/mol creatinine), raising the suspicion of a neuroblastoma. Subsequent analysis of an acidified urine sample confirmed a more than threefold increase in VMA, HVA, normetanephrine, norepinephrine, and 3-methoxytyramine concentrations. In addition, VIP was markedly elevated (1994 ng/L) in a blood sample. The diagnosis of neuroblastoma was confirmed through imaging and histological examination. This case illustrates that chronic diarrhea with metabolic dysregulation (*e.g.* hypokalemia) can be the first and only symptom in patients with VIP-secreting neuroblastoma which can result in delayed diagnosis of neuroblastoma.

## Graphical abstract

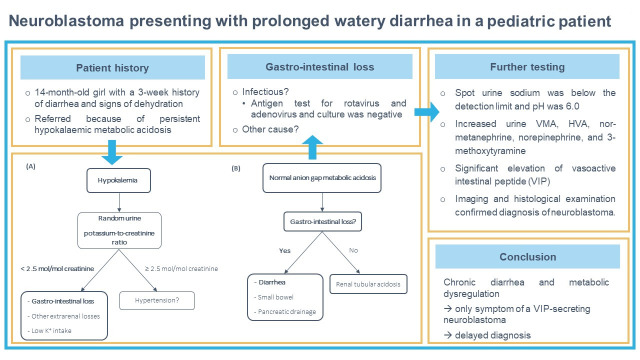

## Highlights

• Vasoactive intestinal peptide (VIP) hypersecretion by malignant tumors is an uncommon cause of intractable diarrhea

• VIP-secreting neuroblastoma is a rare cause of treatment-resistant diarrhea in young children

• Chronic diarrhea with metabolic dysregulation is frequently the only symptom of a VIP-secreting neuroblastoma

## Introduction

Neuroblastomas are the most prevalent extracranial solid tumor in pediatric patients and are derived from immature cells of the neural crest destined for the adrenal medulla and sympathetic nervous system (*1*-*3*). The adrenal chromaffin cells and sympathetic ganglia produce catecholamines (epinephrine (E), norepinephrine (NE) and dopamine (DA)) and their metabolites (metanephrine (MN), normetanephrine (NMN), 3-methoxytyramine (3MT), homovanillic acid (HVA) and vanillylmandelic acid (VMA)) (*4*, *5*). Neuroblastomas are a heterogeneous group of neuroblastic tumors with a widely variable clinical course and degree of differentiation (*6*).

In rare cases, patients with neuroblastoma develop Horner syndrome (disrupted sympathetic innervation on one side of the face with *e.g.* ptosis and miosis), or a paraneoplastic syndrome such as opsoclonus-myoclonus syndrome (development of neuroblastoma-related antineuronal antibodies with involuntary eye movements, muscle jerks, and ataxia) or a syndrome with watery diarrhea, hypokalemia and achlorhydria (WDHA syndrome) related to vasoactive intestinal peptide (VIP) secretion (*5*, *7*-*9*). The VIP is a neurotransmitter and a member of the secretin-glucagon superfamily that can be found in the gastrointestinal tract and the central nervous system in abundance (*9*). It has different effects on the gastrointestinal tract such as water and electrolyte secretion from the pancreas and gut, smooth muscle relaxation of the gut, hormone release from the pancreas, gut and hypothalamus, growth-promoting effect in certain tumors (*e.g.* gastric cancer) and anti-inflammatory activity (*9*, *10*). In a French retrospective study, less than 1% of the patients with a diagnosed neuroblastoma had clinical evidence of VIP secretion (*5*). Patients with VIP-secreting neuroblastoma often exhibit a distinct clinical presentation with chronic diarrhea as one of the first symptoms (*5*). Chronic diarrhea with metabolic dysregulation (*e.g.* hypokalemia) is frequently the first and only symptom which can result in delayed diagnosis of neuroblastoma (*5*).

We describe a patient with prolonged watery diarrhea for 3 weeks, hypokalemia and metabolic acidosis caused by an underlying neuroblastoma.

## Case report

A 14-month-old girl presented to the emergency department of another hospital with watery diarrhea for three weeks with no accompanying symptoms. Upon examination, the patient exhibited lethargy, sunken eyes, and dry mucous membranes. The weight was 9.3 kg (10th percentile). The child was born at 34 weeks and 4 days without any complications. There was no other relevant history or use of medications. Venous blood gas analysis revealed acidosis (pH 7.29), decreased bicarbonate concentration (11.2 mmol/L), severe hypokalemia (2.4 mmol/L), normal anion gap (20 mmol/L), normal lactate (1.6 mmol/L) and urine dipstick analysis a pH of 5.5 and ketonuria (1+) (Table 1, regional hospital). An antigen test for rotavirus and adenovirus (SD Bioline Rota/Adeno rapid, Standard Diagnostics Inc., Kyonggi, Korea) was negative. The girl was referred to our hospital 48 hours after admission for hypokalemic metabolic acidosis with no normalisation after intravenous fluid therapy and KCl supplementation (6 mEq/kg *per* day).

**Table 1:** Point-of-care, urine dipstick analysis, and core laboratory test results at the time of admission to the regional hospital and at the time of admission in our hospital

**Laboratory test (unit)**	**Regional hospital**	**Admission (48h later)**	**Reference range**
Venous blood gas analysis			
pH	**7.29** ↓	**7.29** ↓	7.35-7.43
pCO_2_ (kPa)	3.15	3.53	
pO_2_ (kPa)	**6.52 ↑**	5.39	5.07-5.87
Bicarbonate (mmol/L)	**11.2** ↓	**12.6** ↓	22.0-29.0
Base excess (mmol/L)	**- 14.1** ↓	**- 12.6** ↓	- 2.0 to 3.0
Hemoglobin (g/L)	**113** ↓	**106** ↓	120-160
Sodium (mmol/L)	138	142	136-146
Potassium (mmol/L)	**2.4** ↓	**3.4** ↓	3.5-4.5
Chloride (mmol/L)	**113 ↑**	**119 ↑**	98-106
Anion gap* (mmol/L)	16.2	13.8	10.0-20.0
Calcium ionized pH 7.4 (mmol/L)	1.32 ↑	1.38 ↑	1.15-1.29
Glucose (mmol/L)	-	4.9	3.9-5.8
Lactate (mmol/L)	1.6	1.0	0.5-2.2
Spot urine sample			
pH (dipstick)	5.5	-	
Ketones (dipstick)	**1+**	-	Negative
Venous blood sample (plasma)			
Sodium (mmol/L)	138	138.1	136.0-145.0
Potassium (mmol/L) (serum)	**2.6** ↓	-	3.5-5.1
Potassium (mmol/L) (plasma)	-	3.49	3.45-4-45
Chloride (mmol/L)	110 ↑	**116.1 ↑**	98.0-107.0
Bicarbonate (mmol/L)	10.8 ↓	**9.3** ↓	22.0-29.0
Anion gap (mmol/L)^1^	**20 ↑**	16.2	9.0-20.0
Calcium (mmol/L)	-	2.69	2.25-2.75
Urea (mmol/L)	**11.8 ↑**	2.8	≤ 6.5
Creatinine (µmol/L)	50.4 ↑	**32.7 ↑**	15.9-30.9
eGFR-FAS (mL/min/1.73m^2^)	57 ↓	**72** ↓	≥ 90
Ammonia (µmol/L)	-	**55 ↑**	11-51
Glucose (mmol/L)	-	4.77	3.05-5.55
CRP (mg/L)	-	0.6	≤ 5.0
*Anion gap: ([Na^+^] + [K^+^]) - ([HCO_3_^-^] + [Cl^-^]). eGFR-FAS - estimated glomerular filtration rate - full age spectrum. Na - sodium. K - potassium. Cl - chloride.			

On admission to our hospital, the child was generally in good condition. The examination showed a blood pressure of 114/63 mmHg, a heart rate of 145 beats/minute, a temperature of 36.5 °C, and a weight of 10.4 kg (50th percentile). Stools were watery, not mucoid nor bloody. Further clinical investigation was normal. Venous blood gas analysis upon admission in our hospital revealed acidosis (pH 7.29), decreased bicarbonate concentration (12.6 mmol/L), mild hypokalemia (3.4 mmol/L), normal anion gap (13.8 mmol/L), normal glucose concentration (4.9 mmol/L) and normal lactate (1.0 mmol/L) (Table 1, admission). A parallel venous sample for routine chemistry confirmed the hyperchloremic acidosis (Table 1, admission).

## Considered diagnoses

**Figure 1:**
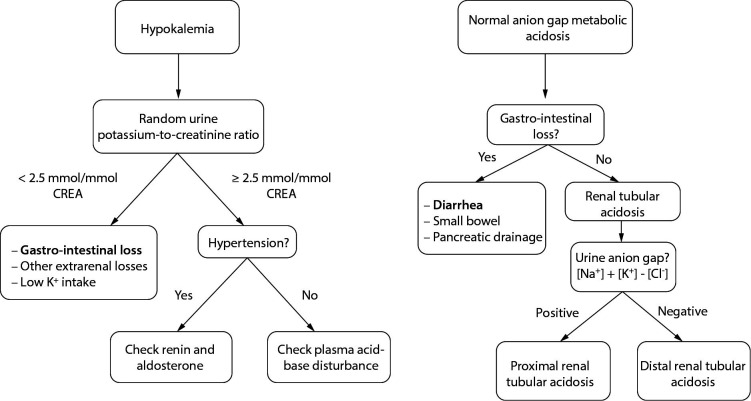
Diagnostic approach to determine the etiology of hypokalemia (left) and normal anion gap metabolic acidosis (right). CREA - creatinine. Na - sodium. K - potassium. Cl - chloride.

The most common causes of normal anion gap metabolic acidosis in young children are gastrointestinal loss of bicarbonate (HCO_3_^-^) resulting from diarrhea or renal loss of HCO_3_^-^ due to the inability to acidify the urine (renal tubular acidosis) (Figure 1) (*11*-*13*). In young children prolonged increased gastrointestinal or renal loss in combination with decreased intake (*e.g.* anorexia) can result in significant hypokalemia (*9*). Given the negative antigen test for adenovirus and rotavirus and persistent hypokalemic metabolic acidosis despite intravenous fluid therapy and KCl supplementation, the patient was referred to our hospital to exclude renal tubular acidosis (RTA) or a possible inborn error of metabolism.

Spot urine sodium 24 hours after admission was below the detection limit suggesting hypovolemia or possible primary hyperaldosteronism (Table 2). Urine potassium concentrations and fractional excretion were also low (< 2.5 mol/mol creatinine, Table 2) suggesting hypokalemia due to prolonged increased gastrointestinal loss in combination with insufficient intake (Figure 1) (*14*). The negative anion gap, an estimate of urinary ammonia, suggests gastrointestinal loss of bicarbonate or proximal renal tubular acidosis (type 2) as a cause of the hyperchloremic metabolic acidosis (*13*, *15*). In distal renal tubular acidosis (type 1), the urine anion gap is positive (*13*, *16*). The urine pH 24 hours after admission was 6.0 without glucosuria, aminoaciduria, or leakage of phosphate (Table 2). While the usual renal response to acute metabolic acidosis with acidemia is to reduce the urine pH to 5.3 or less, the urine pH in case of chronic hyperchloremic metabolic acidosis can be relatively high despite a normal kidney response due to increased ammoniagenesis, especially when associated with hypokalemia and volume depletion (*15*, *17*). Increased urinary ammonia excretion increases the urine’s buffer capacity and urine pH.

**Table 2:** Routine urine laboratory test results in a spot urine sample collected 24 hours after admission to our hospital

**Laboratory test (unit)**	**24 hours after admission**	**Reference interval**
Spot urine sample		
Osmolality (mOsm/kg H_2_O)	444	50-1200
pH (dipstick)	6.0	
Glucose (dipstick)	Negative	Negative
Ketones (dipstick)	Negative	Negative
Creatinine (µmol/L)	6073.2	
Sodium (mmol/L)	**< 10.0** ↓	54.0-190.0
Fractional Na^+^ excretion* (%)	**< 0.04** ↓	< 1
Potassium (mmol/L)	**8** ↓	20-80
Fractional K^+^ excretion* (%)	**1.2** ↓	≥ 10
K^+^/creatinine ratio (mol/mol)	1.32	< 2.5
Chloride (mmol/L)	139	46-168
Urine anion gap** (mmol/L)	< - 121 ↓	- 20 to + 20
Phosphate (mmol/L)	**7.60** ↓	13.00-44.0
Calcium/creatinine ratio (mol/mol)	**3.88 ↑**	0.08-0.75
Total protein (g/L)	**0.26 ↑**	≤ 0.15
*Fractional excretion = ([ion^+^]_urine_ x [creatinine]_plasma_) / ([ion^+^]_plasma_ x [creatinine]_urine_). **Urine anion gap (mmol/L): [Na^+^] + [K^+^] - [Cl^-^]. Na - sodium. K - potassium. Cl - chloride.		

## Further investigations

Amino acids (plasma), acylcarnitines (plasma), and organic acids (urine) were ordered at the time of initial evaluation. Semi-quantitative urine organic acid analysis, reported 48 hours after admission, revealed increased VMA (89 mmol/mol creatinine, reference range ≤ 13) and HVA (21 mmol/mol creatinine, reference range ≤ 19), raising the suspicion of a neuroblastoma. Amino acids (plasma) and acylcarnitines (plasma) were normal. An additional spot urine sample was collected 72 hours after admission for measurement of VMA, HVA, catecholamines and metanephrines. The sample was acidified immediately upon arrival in the laboratory. The results demonstrated a more than threefold increased VMA, HVA, NMN, NE, and 3MT (Table 3).

**Table 3:** Results of catecholamines and metabolites in a spot urine sample and additional tests performed in venous blood to establish the diagnosis

**Laboratory test (unit)**	**Result**	**Reference interval**
Spot urine sample collected 72 hours after admission and acidified in the laboratory		
Creatinine (µmol/L)	2970.3	
Homovanillic acid (mmol/mol creatinine)	110 ↑	≤ 19
Vanillylmandelic acid (mmol/mol creatinine)	89 ↑	≤ 13
Metanephrine (mmol/mol creatinine)	157	≤ 304
Normetanephrine (mmol/mol creatinine)	3788 ↑	≤ 802
3-methoxytyramine (mmol/mol creatinine)	1692 ↑	≤ 203
Epinephrine (mmol/mol creatinine)	10	≤ 51
Norepinephrine (mmol/mol creatinine)	638 ↑	≤ 194
Dopamine (mmol/mol creatinine)	5474 ↑	≤ 901
Additional tests in venous blood		
Plasma renin activity (µg/L/h)	> 40.0 ↑	1.40-7.80
Aldosterone (nmol/L)	1.76	0.56-3.05
Chromogranin A (µg/L)	**93.6 ↑**	6.2-18.0
Neuron-specific enolase (µg/L)	**26.9 ↑**	≤ 16.3
Vasoactive intestinal peptide (ng/L)	1994 ↑	< 101

Additional testing, showed increased renin activity (> 40 µg/L/h) with a normal aldosterone concenttration (1.76 nmol/L), excluding primary hyperaldosteronism, and a major increase of VIP (1994 ng/L) (Table 3). The tumor markers neuron-specific enolase and chromogranin A were also elevated (35.7 µg/L and 93.6 µg/L, respectively) (Table 3). Stool culture for enteropathogens (Salmonella, Shigella, Yersinia and Campylobacter species) was negative after two days.

An abdominal ultrasound unveiled a heterogeneous mass in the right adrenal gland with internal calcifications and a distended abdomen compatible with the suspected diagnosis of a neuroblastoma. Staging was performed according to the International Neuroblastoma Risk Group Staging System (INRGSS) based on clinical criteria and image-defined risk factors to stratify patients before treatment (*18*). An abdominal magnetic resonance imaging (MRI) scan, a metaiodobenzylguanidine (MIBG) scintigraphy and bilateral iliac crest bone marrow aspirate and biopsy were done. No metastases were identified. The tumor was staged as an L2 neuroblastoma with life-threatening symptoms. The pathologist confirmed the diagnosis of ganglioneuroblastoma without N-myc proto-oncogene (MYCN) amplification on fluorescence *in situ* hybridization (FISH) in a biopsy specimen.

Due to the high complexity of the tumor and the proximity of important vasculature (*e.g.* encasement of the coeliac trunk and the superior mesenteric artery), the treating clinicians decided not to perform surgery but to start chemotherapy (carboplatin and etoposide following the LINES protocol). The chemotherapy was stopped after one cycle because no clinical or biochemical response was seen (VIP was 2678 ng/L after one cycle). Everolimus, a protein kinase inhibitor, was administered, but stopped after three weeks due to no effect on the diarrhea and the development of stomatitis despite adequate therapeutic drug concentrations. Other therapy modalities were tried, such as short- and long-acting octreotide. After eight months of profusely secretory diarrhea with hypokalemia, the patient was transferred to Germany for the surgical resection of the neuroblastoma. After the surgery, the watery diarrhea ameliorated and the hypokalemia resolved. The child recovered and no evidence of any residual mass or metastases was seen on abdominal ultrasound or magnetic resonance imaging after 4 months and on abdominal ultrasound after 22 months.

## Laboratory methods

Blood gas analysis was performed using safePICO syringes on ABL90 FLEX (Radiometer, Copenhagen, Denmark) in both hospitals. Heparinized plasma was collected using vacutainer plasma separator tubes (PST, Becton Dickinson (BD), Franklin Lakes, USA) and routine chemistry analytes were measured in heparinized plasma and urine with Cobas 8000 (Roche Diagnostics GmbH, Mannheim, Germany) in both hospitals. Urine pH (dipstick) and ketones were determined with Meditape UC-9A (Sysmex Europe SE, Norderstedt, Germany) at the regional hospital and with Uriflet S (9UB) strip on Aution Max (Arkray, Kyoto, Japan) in our hospital. Osmolality was measured with OM-6050 (Arkray, Kyoto, Japan).

Serum was collected using vacutainer serum separator tubes *(*SST II advance, Becton Dickinson, Franklin Lakes, USA) and EDTA plasma using vacutainer tubes (K2E, Becton Dickinson, Franklin Lakes, USA). Chromogranin A in serum was measured with the Kryptor Compact Plus (ThermoFisher, Waltham, USA) and NSE in serum with the Cobas 8000 (Roche Diagnostics, Rotkreuz, Switzerland). Plasma renin activity in EDTA plasma was measured using the DIAsource Angiotensin I (PRA) radioimmunoassay kit (DIAsource ImmunoAssays, Louvain-La-Neuve, Belgium) and aldosterone in EDTA plasma using the Beckman Coulter Aldosterone kit (Beckman Coulter, Brea, USA). In EDTA plasma VIP was measured with the VIP radioimmunoassay kit (DIAsource ImmunoAssays, Louvain-La-Neuve, Belgium).

In urine, HVA and VMA were measured with a lab-developed method using Alliance e2695 Separations Module and 2465 Electrochemical Detector and free catecholamines (E, NE, DA) and total metanephrines (MN, NMN, 3MT) were measured with a lab-developed method using an I-class separation module and Xevo TQ-XS MSMS detector (Waters, Milford, USA). Both methods are accredited according to ISO15189:2012 by BELAC, the Belgian accreditation organisation. We participate in the external quality assessment scheme Urine chemistry Biogenic Amines of Instand (Düsseldorf, Germany) and the Neuroblastoma Round Robin of UK NEQAS (Birmingham, United Kingdom).

Urine organic acid analysis was performed using a lab-developed method with gas chromatography with mass spectrometric detection (GC-MS, Trace GC with PolarisQ, Thermo Finnigan, Thermo Electron Corporation, Waltham, USA). The method is accredited according to ISO15189:2012 by BELAC and we participate in the external quality assessment schemes quantitative organic acids (urine) and qualitative organic acids (urine) organized by ERNDIM (Manchester, United Kingdom).

## Discussion

Persistent diarrhea is common during childhood and can be caused by varying aetiologies including infection, allergy, and antibiotic use (*19*, *20*). Malignant disease is an uncommon cause of persistent diarrhea and a neuroblastoma presenting with VIP hypersecretion, as in this case report, is a very rare cause of diarrhea (less than 1% of neuroblastomas) (*5*). Verner and Morrison were the first to describe a syndrome with refractory diarrhea and hypokalemia related to a tumor (*21*). The Verner-Morrison syndrome, also called the WDHA syndrome, arises from an overproduction of VIP by a tumor such as a neuroblastoma causing watery diarrhea, hypokalemia, and achlorhydria as symptoms (*5*, *22*). Excessive VIP secretion can result in treatment-resistant diarrhea with electrolyte disturbances (*e.g.*, hypokalemia, hypochlorhydria, hypercalcemia, and hypomagnesemia can develop) and dehydration (*5*, *9*).

Hypokalemia in young children can be due to intracellular redistribution of potassium or true potassium loss. Redistribution can occur following insulin therapy for diabetic hyperglycemia, in the presence of alkalosis, or with the use of beta-adrenergic agonists. Additionally, hypokalemia can arise from true potassium deficits due to reduced intake or increased loss of potassium. Potassium loss may be renal or extrarenal, with diarrhea being the most common cause of extrarenal loss. Renal potassium loss can be due to RTA. All types of RTA result in hyperchloremic normal anion gap metabolic acidosis. Distal RTA (type 1) is caused by defects in distal hydrogen ion excretion. Proximal RTA (type 2) is caused by defects that reduce the capacity to reclaim filtered bicarbonate in the proximal tubule, which could be a possible cause of metabolic acidosis in our patient. Other RTA forms are related to hypoaldosteronism (type 4) or a defect in distal sodium reabsorption (voltage-dependent RTA). Distal and proximal RTA are commonly associated with hypokalemia due to renal potassium wasting, while the other types typically result in hyperkalemia (*23*). In children, the most frequent cause of hypokalemia is excessive gastrointestinal losses, as was the case in our patient (*9*, *24*).

The chronic watery diarrhea resulted in a gastrointestinal loss of bicarbonate, an important blood acid buffer, and metabolic acidosis. The patient had elevated chloride blood concentrations. Chloride plays a crucial role in intracellular and extracellular acid-base regulation. In this case, both plasma and urine anion gaps were reduced, which is indicative of diarrhea and proximal RTA (*13*, *15*, *23*). The hypokalemic, hyperchloremic normal anion gap metabolic acidosis observed in this case was explained by intestinal bicarbonate and potassium loss due to chronic diarrhea. The decreased urine anion gap (AG) helped to exclude distal renal tubular acidosis (increased urine AG) (*15*, *16*).

Proximal RTA is often linked to a generalized dysfunction of the proximal tubules, manifesting as Fanconi syndrome. However, in this case, the diagnosis of proximal RTA was less likely, given the lack of glucosuria, hyperphosphaturia, and aminoaciduria, which are characteristic of Fanconi syndrome. Furthermore, proximal RTA due to drug use or metabolic disease was ruled out based on the patient’s medical history (*23*).

The diagnosis of neuroblastoma is based on a combination of biochemical analyses, imaging and histological confirmation. Biochemical screening traditionally involves the measurement of vanillylmandelic acid (VMA) and homovanillic acid (HVA) concentrations in a 24-hour urine sample (*25*). However, due to the suboptimal diagnostic performance of this approach (sensitivity 73%-92% with a specificity of 96%-100%), alternative catecholamine metabolites in urine and plasma have been investigated (*26*). In a study conducted by the SIOPEN Catecholamine Working Group in 2023, the area under the receiver-operating-characteristic curve of a panel of eight-catecholamine metabolites (VMA, HVA, MN, NMN, 3MT, E, NE, DA) was significantly higher compared to only VMA plus HVA (*25*). VMA, HVA, DA, 3MT, and NMN (which were elevated in this case) were the most sensitive catecholamine metabolites and were positive in more than 60% of patients (*25*). In contract, NE (elevated in this case) was only positive in one third of the patients and NM and E (normal in this case) in less than 20% of patients (*25*). The low sensitivity of E and NM can be explained by the lack of phenylethanolamine-N-methyltransferase (PNMT) expression in neuroblastomas (*27*). Typically, an ultrasound is part of the initial examination performed when an abdominal mass is suspected in pediatric patients. In this patient, an abdominal ultrasound revealed a mass consistent with neuroblastoma, which was subsequently confirmed by histopathological examination. Staging included imaging with MRI, a MIBG scan for possible bony metastases, and bilateral iliac crest bone marrow aspirate and biopsy.

Neuroblastomas have a widely variable clinical presentation and complex biology depending on the site of origin of the tumor. The immature origins of neuroblastomas explain the variety of possible locations along the sympathetic nervous system. Most tumors arise in the abdomen, especially at the level of the adrenal gland (*1*). Neuroblastomas secreting VIP generally behave biologically favourably. MYCN amplification and metastases are rarely seen (*1*, *5*, *22*). MYCN is a commonly used genomic biomarker to give additional information about the tumor behaviour and prognosis and MYCN amplification is linked to poorer prognosis (*3*, *18*).

In general, the initial management of an L2 neuroblastoma without MYCN amplification involves chemotherapy with or without surgical intervention or, in selected cases, a watchful waiting approach (*8*, *28*). However, according to the 2021 standard clinical practice recommendations for low-risk neuroblastoma issued by the European Society for Pediatric Oncology (SIOPE), patients experiencing diarrhea due to VIP secretion from neuroblastic tumors do not respond to chemotherapy and require definitive surgical intervention (*28*). Chemotherapy (*e.g.*, etoposide and carboplatin) can be used in patients with a low-risk neuroblastoma who have image-defined risk factors and/or life-threatening symptoms to stabilize their condition. This was, however, unsuccessful in our patient. Everolimus was also trialled (without success) because of the possible antisecretory and antitumoral effect shown in pancreatic neuroendocrine tumors secreting VIP (*29*). The SIOPE guideline states that surgical resection can be considered in patients when image-defined risk factors (*e.g.* tumor wrapped around a major blood vessel) are no longer present (*28*). As an antisecretory short-term treatment, somatostatin analogues, such as octreotide, are frequently used in pancreatic VIPoma (*29*). Somatostatin acts as an inhibitor of the secretion of different peptides and amines in the gastrointestinal system by binding to the somatostatin receptors on the surface of tumor cells (*30*).

In conclusion, chronic diarrhea with metabolic dysregulation (*e.g*. hypokalemia) can be the first and only symptom in patients with a VIP-secreting neuroblastoma. Physicians and laboratory medicine professionals should include neuroblastoma in the differential diagnosis of young children with treatment-resistant diarrhea to avoid delayed diagnosis.

## Data Availability

All data generated and analyzed in the presented study are included in this published article.
